# Effect of pelvic laparoscopic implantation of neuroprosthesis in spinal cord injured subjects: a 1-year prospective randomized controlled study

**DOI:** 10.1038/s41393-021-00693-7

**Published:** 2021-08-24

**Authors:** Helge Kasch, Uffe Schou Løve, Anette Bach Jønsson, Kaare Eg Severinsen, Marc Possover, Søren Bruno Elmgreen, Axel Forman

**Affiliations:** 1 Spinal Cord Injury Centre of Western Denmark, Department of Neurology, Viborg Regional Hospital, Viborg, Denmark; 2 Department of Clinical Medicine, Aarhus University, Aarhus, Denmark; 3 Department of Surgery, Viborg Regional Hospital, Viborg, Denmark; 4 Possover International Medical Centre, Zürich, Switzerland; 5 Danish Defense Medical Command, Aarhus, Denmark; 6 Department of Obstetrics and Gynecology, Aarhus University Hospital, Aarhus, Denmark

**Keywords:** Spinal cord diseases, Randomized controlled trials

## Abstract

**Study design:**

1-year prospective RCT.

**Objective:**

Examine the effect of implantable pulse generator and low-frequency stimulation of the pelvic nerves using laparoscopic implantation of neuroprosthesis (LION) compared with neuromuscular electrical stimulation (NMES) in SCI.

**Methods:**

Inclusion criteria: traumatic spinal cord injury (SCI), age 18–55 years, neurological level-of-injury Th4–L1, time-since-injury >1 year, and AIS-grades A–B. Participants were randomized to (A) LION procedure or (B) control group receiving NMES. Primary outcome measure: Walking Index for Spinal Cord Injury (WISCI-II), which is a SCI specific outcome measure assessing ability to ambulate. Secondary outcome measures: Spinal Cord Independence Measure III (SCIM III), Patient Global Impression of Change (PGIC), Penn Spasm Frequency Scale (PSFS), severity of spasticity measured by Numeric Rating Scale (NRS-11); International Spinal Cord Injury data sets-Quality of Life Basic Data Set (QoLBDS), and Brief Pain Inventory (BPI).

**Results:**

Seventeen SCI individuals, AIS grade A, neurological level ranging from Th4–L1, were randomized to the study. One individual was excluded prior to intervention. Eight participants (7 males) with a mean age (SD) of 35.5 (12.4) years were allocated to the LION procedure, 8 participants (7 males) with age of 38.8 (15.1) years were allocated to NMES. Significantly, 5 LION group participants gained 1 point on the WISCI II scale, (*p* < 0.013; Fisher´s exact test). WISCI II scale score did not change in controls. No significant changes were observed in the secondary outcome measures.

**Conclusion:**

The LION procedure is a promising new treatment for individuals with SCI with significant one-year improvement in walking ability.

## Introduction

Spinal cord injury (SCI) is a devastating life-event that may leave the individual with a lifelong severe disabilities, and a need for assistive devices and a restricted capacity in daily living [1]. Early neurorehabilitation is associated with functional improvements in the early stages after SCI, but the long-term effects of neurorehabilitation are more defensive to prevent further deterioration [2–4].

Internal spinal and neural stimulation through implantable pulse generators (IPG) to restore motor and sensory function together with autonomic control has gained an increased interest in recent years, but so far data on the efficacy of this approach are sparse [5].

Laparoscopic implantation of neuroprosthesis (the LION procedure) has shown early and long-term effects in retrospective case series for regaining motor and sensory function in paraplegia after SCI [6, 7]. The present study fulfills an urgent and unmet need for randomized, prospective controlled studies on the effect of LION in spinal cord injured subjects.

In the present randomized controlled study, we, therefore, investigated whether the LION procedure and the subsequent neurostimulation and training regimen in individuals with chronic traumatic complete (AIS grade A) thoracolumbar SCI with spastic paraplegia would lead to weight-bearing standing as compared to standard training with neuromuscular electrical stimulation (NMES) [8]. Additionally, we further investigated the safety of the LION procedure and the subsequent neurostimulation.

## Methods

The study protocol (sensory and motor control in spinal cord injury after laparoscopic neuroprosthetic implant to pelvic lumbosacral nerves) was approved by the Central Denmark Region Committees on Health Research Ethics (ref. no. 1-10-72-409-17, renewal: 1-16-02-129-16) and registered on EudraCT (ref. no. 2017-003433-28) and clinicaltrials.gov (NCT03441256) before inclusion of the participants. The study and technical devices were also approved by the Danish Medicines Agency (Ref.no. 2017080415, CIV-17-08-020985).

### Study design

This clinical investigation was a 1-year prospective randomized controlled trial with two parallel arms: firstly, an intervention group receiving the LION procedure and subsequent neurostimulation and secondly, an active control group receiving long-term home-based NMES [8]. After completion of the present study, the patients in the control group were offered the LION procedure.

Inclusion criteria were traumatic spinal cord injury at least twelve months before enrollment into the study protocol. Participants had SCI graded as AIS A or B, age between 18 and 55 and the injury level from Th4 to L1. Exclusion criteria were pregnancy or planning of pregnancy during the study period, and severe somatic or psychiatric disease, which could jeopardize compliance to the study protocol. Previous surgery with major risk of pelvic fibrosis and the use of other implanted medical devices (e.g., baclofen or insulin pumps, pacemakers) were also considered exclusion criteria.

Following screening for inclusion and exclusion criteria, eligible subjects were randomized at one time point. Screening for inclusion, including AIS classification, was performed by a trained specialist neurologist. Neither subjects nor investigators were blinded to group allocation and randomization was performed by means of the *ralloc* command in the statistical software package Stata 15.0^TM^ (StataCorp, Texas, USA) based on subject ID. Subject exclusion due to secondary ineligibility arisen during implantation was managed by subsequent rolling inclusion of next eligible subject.

#### Participants

Potential participants were identified in a local clinical quality database [DK: “Vestdansk Database for Rygmarvsskader” (Ref.no. 2012-41-0572)] containing details of individuals who were previously admitted at the Spinal Cord Injury Centre of Western Denmark (SCIWDK). Eligible subjects were contacted by postal mail and the subjects were invited together with their close relatives to information meetings at SCIWDK concerning study participation.

### Intervention

#### Medical devices

We used the Precision Spectra IPGTM (dimensions: 55 × 46 × 11 mm, volume: 21 ml) from Boston Scientific Corporation (BSC), Marlborough, Massachusetts, USA, with four linear ST electrode leads of 50 cm or 70 cm, the FreeLink remote control system and the standard wireless charging system. Programming was performed using the Clinician Programmer^TM^ and the associated software, BionicNavigator 1.2. BSC’s Precision Spectra IPG, and the associated leads are CE and FDA approved for implantation in human for treatment of chronic, intractable pain.

Laparoscopic surgery was performed at the Department of Surgery (USL, AF, MP) in accordance with previous studies [9, 10]. In summary, the pelvic portion of the sciatic and the femoral nerves were exposed and the separate 8-electrode arrays were then placed longitudinally to the sciatic and the femoral nerves on both sides. Subsequently, the leads were tunneled to the abdomen, where the IPG was placed subcutaneously. Intraoperative electrical stimulation assured the correct lead placement and lead functioning.

#### Neurostimulation and training protocol

Three weeks after the participants allocated to the LION procedure had their implantation performed, the stimulation was initiated with continuous neurostimulation using all four leads with the lowest possible current intensity needed for subclinical skeletal muscle contraction (frequency: 5–10 Hz. pulse width: 50–150 μs, current intensity: variable).

Approximately 6 weeks after the LION procedure, the training programs were initiated. Training programs consisted of stimulation for 20–30 min during home training sessions every other day. Current intensities needed in the range from minimal to maximal knee extension via femoral leads and gluteal contractions via sciatic leads were used (frequency: 30–60 Hz, pulse width: 50–150 μs, current intensity: variable, cycle: 30 s on/10 s off). Participants were instructed to cooperate with the stimulation, and voluntarily trying to extend the knee during the knee extension training program. The training session should cease when there was no longer a visible movement of the lower leg or the gluteal muscles. If the participants developed sufficient muscle strength to support standing at three months follow-up or subsequently, stand was allowed with concomitant stimulation on all four leads. The principal investigator and two experienced physiotherapists instructed the participants to stand up from the wheelchair using the stand stimulation program in either parallel bars or a standing table. If the participants had the necessary aids available, stand training was allowed as once a day home training as well. Walking was only attempted at follow-up visits with the support of two physiotherapists in parallel bars.

In the control group, NMES was initiated using a standard wired 4-channel NMES device (Chattanooga Physio, DJO, Lewisville, Texas, USA). Subjects were instructed to self-administer a preinstalled program developed for disuse atrophy 2–3 times a week for 20–30 min using currents sufficient to elicit visible muscle contraction. NMES stimulation included the gluteal and the quadriceps muscle groups. A written instruction was handed out with guidance by the principal investigator for the home-based training.

### Outcomes

The primary endpoint was change in subjects’ ability to ambulate, which was assessed at baseline and at follow-up using the Walking Index for Spinal Cord Injury (WISCI II). WISCI II is an ordinal scale ranging from 0 to 20. SCI individuals with WISCI II score amounting to 0 are unable to stand and/or participate in assisted walking. SCI individuals with WISCI II score 1, are enabled to ambulate in parallel bars, with braces and physical assistance of two therapists, at a distance less than 10 meters. WISCI II scores from 2 to 20 all correspond to increasing ambulatory capacity. A score of 20 represents individuals who are enabled to ambulate with no devices, with no braces and no physical assistance, at a 10-meter distance [11, 12]. In the present study, only ankle-foot orthoses were used and allowed during the performed measurements.

Furthermore, at baseline and one-year follow-up a range of secondary endpoints were assessed; Spinal Cord Independence measure III [13] (SCIM III), Patient Global Impression of Change (PGIC) [14], self-reported spasm frequency measured by Penn Spasm Frequency Scale (PSFS) [15] self-reported severity of spasticity measured by Numeric Rating Scale (NRS-11) [1]. International Spinal Cord Injury data sets-Quality of Life Basic Data Set (QoLBDS) [16] and the development in pain as measured by the Brief Pain Inventory (BPI) [17, 18].

Adverse events, procedural and surgical factors were assessed in the LION group, but are published elsewhere in detail [19].

#### Statistical analysis

The sample size of the present study was calculated based on data reported by Possover et al. who found a change in mean WISCI II score with electrical stimulation turned on of 8.5 (SD 3.0) [6]. The present study was powered to show a delta effect in the active group of 3 points in average. We, therefore, estimated a sample size of 10 participants in each group (Power 83%, alpha 0.05).

When there is an improvement of one level on the WISCI II (levels 0–20) it is considered clinically meaningful [12, 20, 21]. Due to the small treatment arms the main outcome parameter was dichotomized into improved WISCI II level (Yes/No). The Fischer’s Exact test, one-sided was applied. For further analysis on normally distributed secondary outcome measures as assessed by the Shapiro–Wilk test, Student´s *t*-test was used. In case of skewness in data distribution a Mann–Whitney–Wilcoxon test was applied.

## Results

**Fig. 1:**
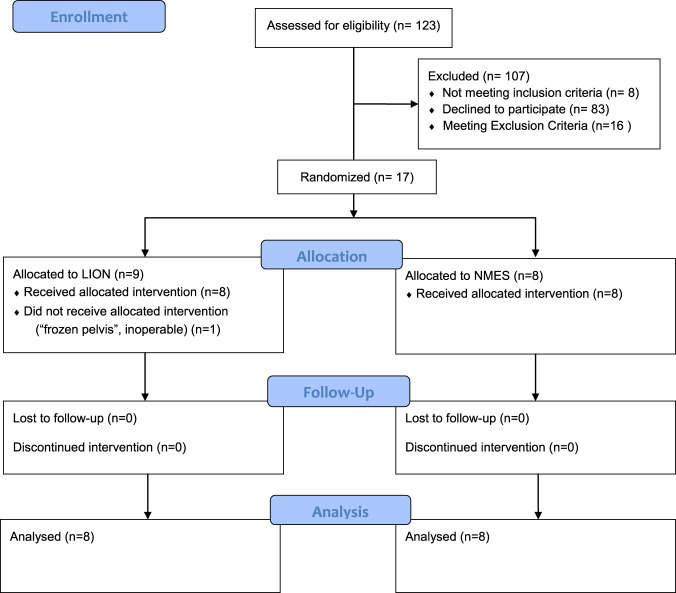
Flow of participants throughout the study. Randomized controlled study with LION procedure and neurostimulation with one-year follow-up in SCI.

Due to the lack of further eligible patients at the end of the scheduled study period, only seventeen SCI patients were included and randomized to either the LION procedure (*n* = 9) or to the control group receiving NMES (*n* = 8). One patient in the LION group had severe pelvic fibrosis at laparoscopy, precluding application of the electrodes. Consequently, this participant was excluded (Fig. 1).

Demographic details, participant characteristics, and clinical properties are provided in Tables 1 and 2.

**Table 1:** Demographics and initial group characteristics.

	LION (*n* = 8)	Control (*n* = 8)
Age (Mean ± SD)	35.5 (12.4)	38.8 (15.1)
Gender, male%	7 (87.5)	7 (87.5)
Single/married or common-law	4/4	5/3
Job description		
Disability pension/unemployed	3	5
Working	5	3
Time since injury (year, mean ± SD)	12.39 (9.47)	16.30 (9.03)
AIS Grade A	8/8	8/8
NN-level (thoracic/lumbar)	8/0	7/1
WISCI Baseline (0–20) (median (range))	0 (0;12)	0 (0;0)
ISCoS QoLBDS1, general QoL	8.12 (0.99)	7.5 (1.4)
ISCoS QoLBDS2, physical health	7.12 (1.55)	6.88 (2.03)
ISCoS QoLBDS3, psychological health	8.63 (1.06)	8.13 (0.64)
SCIM Baseline (Total 0–100) (mean ± SD))	70.38 (2.77)	66.75 (5.70)
Brief pain inventory average (VAS 0–10) (mean ± SD)	0.38 (1.1)	1.00 (1.60)
Brief pain inventory least (VAS 0–10) (mean ± SD)	0.13 (0.35)	0.13 (0.35)
Brief pain inventory worst (VAS 0–10) (mean ± SD)	1.00 (2.82)	2.5 (3.8)
Penn spasm frequency (0–4) (mean ± SD)	1.50 (1.20)	1.38 (0.91)
Spasticity severity (NRS-11) (mean ± SD)	2.50 (2.51)	2.50 (3.01)

*LION* laparoscopic implantation of neuroprosthesis.

**Table 2:** Participant Characteristics.

Age	Gender	AIS	Neurological	Time since	WISCI II	WISCI II	WISCI II	SCIM III	SCIM III
		Grades	Level	Injury (y)	Initial	3 mth	1-year	Initial	1-year
*LION GROUP*									
45	Male	A	Th4	22.8	0	1	1	72	74
26	Female	A	Th6	2.4	0	0	1	67	65
29	Male	A	Th10	2.7	12	12	12	72	74
45	Male	A	Th10	16.2	0	0	0	65	63
47	Male	A	Th6	26.1	0	0	0	71	63
34	Male	A	Th4	16.9	0	1	1	72	72
23	Male	A	Th5	2.4	0	1	1	72	74
28	Male	A	Th5	9.3	0	0	1	72	74
*NMES GROUP*									
30	Male	A	Th7	8.4	0	0	0	71	72
47	Male	A	Th10	16.8	0	0	0	71	54
34	Male	A	Th4	17.6	0	0	0	72	62
36	Male	A	Th8	9.1	0	0	0	72	63
31	Male	A	Th11	2.9	0	0	0	65	67
42	Female	A	Th5	24.3	0	0	0	69	67
46	Male	A	Th5	26.5	0	0	0	71	71
50	Male	A	L1	24.4	0	N/A	0	72	73

*AIS* ASIA impairment scale.

At baseline there was no significant difference in demographics or severity of SCI in the two groups. Time since injury, AIS grading, neurological level of injury and WISCI II and initial SCIM scores were also similar (Table 1).

### Primary outcome measure

At one year follow-up, the WISCI II score increased from 0 to 1 in 5 of 8 participants in the LION Group whereas, there was no change in the control group, *p* = 0.013 (Fisher´s exact test). However, this increase was already achieved at 3 months follow-up in 3 of 8 LION Group participants and at 6 months in 5 of 8 participants (Table 2).

One of the LION group participants was partly mobile at inclusion (level 12 on the WISCI scale) but this subject did not improve further at 1-year follow-up (Table 2). By ASIA definition [22] this particular participant was scored as AIS A because of absent voluntary anal contraction and deep anal pressure sensation.

### Secondary outcome measures

There was no change in SCIM III, PGIC, PSFS, NRS-11, QoLBDS, or BPI measured in the LION group or in the control group.

### Adverse events

Four of 8 SCI participants undergoing LION procedure complained of post-operative shoulder pain that resolved on Over-The-Counter medication within the first 2 weeks. Three of 8 participants complained of gastrointestinal problems (constipation, abdominal pain, nausea) which had resolved within 1-week post-operatively [19]. Three of 8 participants in the LION group developed increased spasticity in lower body/extremities during the first 2 weeks after the continuous stimulation was initiated. However, spasticity was resolved or restored to preoperative levels after a few weeks. In one subject the IPG migrated within 2 months after operation and tilted which made recharging of the IPG impossible. Subsequently, the IPG seized functioning due to battery drainage. After repositioning of the IPG within 2.5 months post-operatively during an outpatient clinic operative procedure, again the IPG was fully functioning and the training program was resumed [19].

## Discussion

In the present RCT the main results revealed a statistically and clinically significant improvement in ambulation as measured by the WISCI II scale in the LION group. No change was observed in the control group. Furthermore, there were no changes in the secondary outcome measurements in either of the groups.

The WISCI II score improvement in the LION group is modest and none of the participants achieved more than one level of improvement at one-year follow-up. This result does not fully comply with a previous published case series reporting data covering 10-years’ experience in a heterogeneous population of 28 SCI individuals with AIS A-C undergoing LION procedure [7]. In the present study participants were randomized to intervention and all participants were AIS categorized by a trained neurologist at inclusion, and the population was homogeneous with regards to AIS grade and exposure to a traumatic SCI at least 12 months before enrollment in the study. Training in the LION-group was homebased, hence there was lack of intensive in-hospital rehabilitation, and follow-up was short which may explain the relatively small change in WISCI II score reported in the present study. However, a WISCI II score change of one is still considered as clinical meaningful [12, 20, 21].

In future studies, long term follow-up and standardized individualized intensive rehabilitation are needed to further unfold the potential of the LION procedure and the impact on walking capacity. Furthermore, evaluation of the impact of the LION-procedure on participant activity and participation is important, thereby, enhancing the clinical relevance of the LION procedure to the SCI individuals.

In future studies, it may be beneficial to include measurements of muscle strength and endurance since individuals with SCI classified as AIS A, having no motor function of the lower extremities, can achieve a change from 0 to 1 point on the WISCI II simply by using long braces fixating the knees in an extended position. Consequently, individuals with AIS A will be able to use the trunk muscles and upper extremities to produce a circumduction gait pattern. However, in the present study, only ankle-foot orthosis was used eliminating this option of gait. By including measurements of muscle strength in future studies, a correlation calculation between lower extremity muscle capacity and ambulation is possible. Thereby, providing an opportunity to substantiate a possible effect of the LION procedure on motor function in individuals with SCI.

### Study limitations

The present study is the first prospective randomized controlled study on the LION procedure in SCI. However, the study has some methodological limitations. A successful blinding of the principal investigator was not fully possible. After one patient was found inoperable at the operation theatre (frozen pelvis), an extra participant had to be included to the LION group, which was performed after all eligible SCI participants were randomized using STATA^TM^.

Another limitation is the use of WISCI II as an outcome measurement. Assessment of the ambulatory function is of major importance after the LION procedure. However, in the present study design with a NMES control group only a relatively short-term follow-up of the groups was for ethical reasons possible. Detection of even small changes in ambulatory function in patients with a complete SCI are highly relevant at a short-term follow-up, but these tiny improvements are difficult to measure when using an ordinal scale such as WISCI II. Previously, however, WISCI II has been described as a valid and reliable tool for assessment of ambulatory function in individuals with SCI [11, 12].

A third limitation is the lack of physiotherapeutic guidance and training as a part of the intervention. In the protocol of the present study, the LION participants all performed home-based training following a brief introduction to the use of the IPG device and remote control and recharging system while systematic physiotherapeutic treatment was not offered. Systematic physiotherapeutic treatment may be needed to fully unleash the potential of the LION procedure and the subsequent neurostimulation.

## Conclusion

This is to our knowledge the first 1-year prospective randomized controlled trial that examines the outcome of the LION procedure in a homogeneous population of SCI individuals. This study reports a small but clinically relevant and statistically significant increase in the participants ambulation abilities as compared to results obtained with standard external neurostimulation. In the future the LION procedure may offer a treatment option in SCI, however, there is a need for further validation of the present results in studies including systematic rehabilitation effort and longer duration of follow-up with outcomes addressing patient activity and participation.

## Data Availability

Deidentified data may be available upon request and approval from the Danish Data Protection Agency. Unidentifiable data may available upon request and approval from the Danish Protection Agency.
